# Graph-theoretic analyses of saturation fraction of repulsive dopants in solid solutions

**DOI:** 10.1038/s41598-025-30829-1

**Published:** 2026-03-12

**Authors:** Atsushi Kubo, Yosuke Abe

**Affiliations:** Japan Atomic Energy Agency, 2-4 Shirakata, Tokai-mura, Ibaraki 319-1195 Japan

**Keywords:** Materials science, Physics

## Abstract

Short-range order (SRO) of dopant atoms in alloys or solid solutions is one of the most essential factors for materials design. In various alloy materials, dopant atoms repel each other, which causes a non-neighboring SRO and results in a substantial effect on their material properties. The fraction of repelling dopants should have an upper bound to satisfy the non-neighboring placement, where dopants are, as it were, saturated. Such “saturation fraction” is expected to play an important role in composition design for alloys. However, no comprehensive understanding has been established thus far for the saturation fraction of repulsive dopant elements despite its practical importance. Here we show that the saturation fraction of repulsive dopant can be described universally by several simple parameters regarding the lattice structure. We conducted a series of stochastic simulation and mathematical analysis for random packing of repulsive dopant in lattice systems for the purpose of predicting the saturation fraction. The mathematical model, which is based on random graph, successfully reproduced the basic trend of the saturation fraction for a variety of lattice structures. The present analyses can provide new insights into composition design for various kinds of alloys such as multi-principal element alloys.

Alloying is one of the most common methods to improve materials’ properties. Not only metals for mechanical application, but also functional materials (such as semiconductors) are often alloyed for optimizing their electric properties, magnetic properties, thermal properties, etc. Moreover, recent researches established a new type of alloy called multi-principal element alloys (MPEAs)^1–4^.

This study deals with substitutional solid solutions as a typical class of alloys, where each site (in a given lattice) is occupied by either a base element or a dopant (additive) element. Many MPEAs also belong to this category, although base elements and additives are no longer distinguished in them. In general, the position of dopant atoms in alloys is not completely random but has a certain trend called short-range order (SRO). SRO is thought to cause a substantial impact on the mechanical and function properties and has been intensively investigated thus far by experimental and theoretical/computational approaches ^5–9^.

Some dopant elements in an alloy may interact in a repulsive manner with each other; i.e., they tend to be placed in order not to be neighboring with the same type of elements. For example, a recent experimental study of a vanadium-cobalt-nickel (V-Co-Ni) ternary alloy observed an evident lack of the V-V pair ^10^. Such elements tend to avoid the same type of element as its neighbor and to be located on the non-neighboring sites in lattice, resulting in formation of a sort of SRO.

From these facts regarding repulsive dopant elements, the following question naturally arises: How dense can dopant atoms be theoretically, if they are randomly placed to the lattice sites under the condition that they are not neighboring to each other? This problem itself is a purely mathematical issue related to the graph theory (since a lattice is a sort of graph); Or, we can regard (if we prefer so) this problem as a variation of random packing problems such as random close packing of circles or spheres ^11,12^. In the present case, we deal with a random packing process on lattices, i.e., discrete spaces where adjacency between points (lattice sites) is given.

Besides purely scientific interest, this problem can be of practical interest in materials design. One of the most important examples is ferritic stainless steel, which is a solid solution-type alloy of iron (Fe) and chromium (Cr), with the body-centered cubic (BCC) lattice structure. It is known that Cr-rich precipitates are formed under certain conditions ^13,14^. Owing to brittle nature in the Cr-rich precipitates, they may cause a fatal problem on the reliability of machinery systems. In addition, neutron irradiation promotes the formation of Cr-rich precipitates, and thus it is an urgent issue to suppress it, especially for the nuclear applications. An isolated (or dilute) Cr atoms in Fe matrix interact repulsively with each other because of spin repulsion ^15,16^. On the other hand, if a sufficient amount of Cr atoms are concentrated locally, they can be stabilized by forming an antiferromagnetic order, resulting in formation of a Cr-rich precipitate. Then, the above-mentioned question about the maximum density (or fraction) has a practical importance in the context of materials design, because it is expected that formation of Cr-rich precipitates is promoted if Cr atoms exist as densely as they are inevitably neighboring with each other. In addition, recent investigation for the Fe-Cr-Al alloys, where aluminum (Al) atoms are added to suppress formation of Cr-rich precipitates, reported avoidance of the Al-Al pair ^17,18^. The SRO of Al atoms is expected to play an important role in suppression of Cr-rich precipitates.

Another example is the group-IV semiconductor alloys such as germanium-tin (Ge–Sn) ^19–21^ , silicon-tin (Si–Sn) ^22^, silicon-germanium-tin (Si–Ge–Sn) ^23–25^ and germanium-lead (Ge-Pb) ^26^ solid solutions, whose electronic properties are affected by SROs between dopant atoms. Ge-Sn solid solutions are promising for various optical applications, thanks to a direct band gap in the mid-infra red region. The band gap of the Ge-Sn solid solution system is known to decrease with increasing fraction of Sn. There is a discrepancy between the threshold fraction by experiment and that by the first principles calculation for a random solid solution assumption. This discrepancy was explained by another calculation introducing SRO, which indicates that a lack of the Sn-Sn pair plays an important role in the electronic properties of the whole Ge-Sn system. This history also implies difficulty in directly observing SROs of repulsive atom pairs or distinguishing SROs from random distribution. Also in the case of the Ge-Pb system, the effect of avoidance of the Pb-Pb pair on the electronic properties was reported ^26^.

Moreover, many kinds of multi-component alloys are affected by such repulsive SROs, which is confirmed from the probability distribution functions, the Warren-Cowley parameter ^27,28^, or other related parameters ^29–33^. Nevertheless, a repulsive SRO is difficult to observe in an experiment because its effect is not always visible in an explicit way, unlike the attractive interaction between atoms with the same element type (presumably resulting in explicit segregation or phase separation ^7,34^).

In this study, we investigate the statistical capacity (namely, saturation fraction) of dopant elements that have a tendency of mutual repulsion. First, we introduce the basic procedure to evaluate the saturation fraction and conducted a series of random packing simulation for various lattice structures according to that procedure. Next, we formulate a mathematical model of the random packing procedure for the purposes of simplifying and generalizing the problem and for deriving universal insights. After validation of the developed mathematical model, comparison between the simulation and mathematical model results reveal a universal trend in the saturation fraction of non-neighboring dopant elements.

## Results

### Random packing process on lattice

**Fig. 1:**
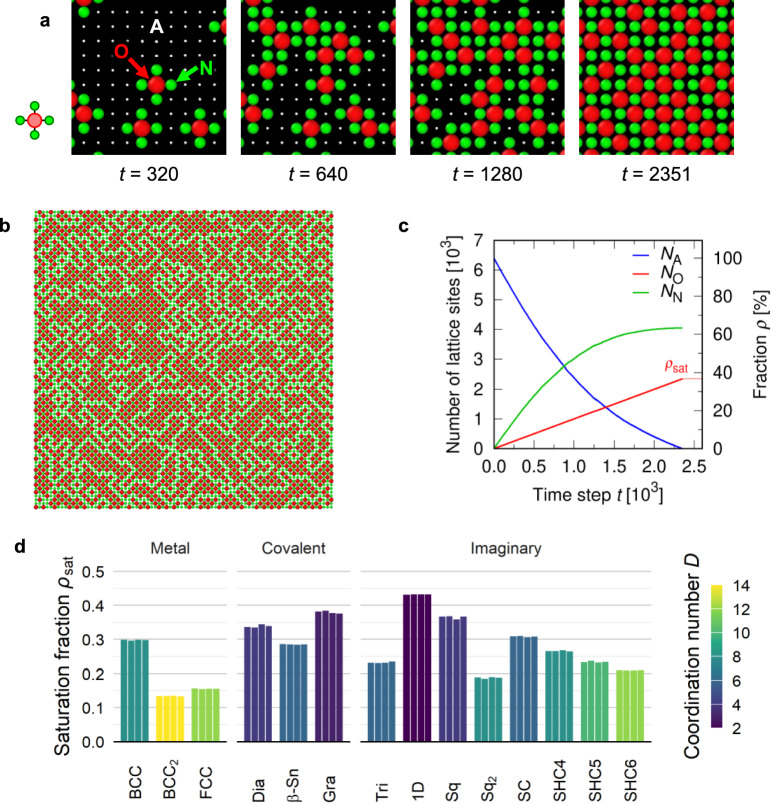
Random packing simulation results for lattice models. (**a**) Temporal evolution of the configuration (enlarged at a fixed position) for the simple square lattice within first nearest neighbor (Sq model). The O-, A-, and N-sites are represented by red sphere, white dot, and green sphere, respectively. (**b**) Entire configuration of Sq model at the final state ($t = 2351$). (**c**) Numbers of O-, A-, and N-sites as functions of time in Sq model. The fraction of the O-site at the final state is saturation fraction ${\rho _\textrm{sat}}$. (**d**) Saturation fraction for all lattice models. Four bars for each case represent the results for four trials.

Figure 1a describes the system to be discussed in this study, and a video of the process is available as Supplementary Video 1a. Each lattice site is either (i) occupied by a dopant atom, (ii) neighboring with a dopant atom, or (iii) available as a potential dopant site. Below we call those three types of sites “O-site”, “N-site”, and “A-site”, respectively. Note that the N- and A-sites are regarded as occupied by base elements. When a dopant atom is added into the system, its site is randomly selected from the A-sites. This can be regarded as a conditional probability problem, where we consider the random placement under the non-neighboring condition.

The algorithm of the process to randomly place dopant atoms to lattice sites (hereafter the “random packing process”) is described as follows:

1. The states of all lattice sites are initialized as the A-sites. In other words, the whole system is pure base material, and any of the atoms can be replaced by a dopant atom.
2. At each time step, one lattice site is randomly selected from the A-sites with an equal probability. The selected A-site is changed to the O-site. This process corresponds to increasing the fraction of the dopant element. Once an O-site is set, its position is unchanged, and no migration is assumed.
3. The adjacent lattice sites is changed to N-sites.
4. Steps 2 and 3 are repeated until all the A-sites are changed to O- or N-sites.

At the final state, dopant is statistically saturated in the lattice, and the fraction of the O-site at saturation, ${\rho _\textrm{sat}}$, is the property that we want to evaluate. The aim of this study is to establish a general theoretical model to estimate ${\rho _\textrm{sat}}$ for an arbitrary lattice structure.

### Numerical simulation of random packing on lattice

Based on the algorithm shown in the previous section, Stochastic simulations of the random packing process were conducted for individual lattice structures. We calculated the temporal evolution of the numbers of the O-, A-, and N-sites (denoted by ${N_\textrm{O}}$, ${N_\textrm{A}}$, and ${N_\textrm{N}}$, respectively) to evaluate the saturation fraction ${\rho _\textrm{sat}}$. Hereafter, the fractions of the O-, A-, and N-sites are denoted by ${\rho _\textrm{O}}:={N_\textrm{O}}/N$, ${\rho _\textrm{A}}:={N_\textrm{A}}/N$, and ${\rho _\textrm{N}}:={N_\textrm{N}}/N$, respectively, where *N* is the number of the lattice sites in the system. The types of the examined lattice structures, their abbreviations, and the detail of the simulation models are shown in Methods. We examined 14 cases (Table 1) with various lattice structures and neighboring conditions, and conducted four independent stochastic trials for each case.

**Table 1:** Examined lattice models.

Lattice	Symbol	Dimension	Neighbor	*D*	*N*	Girth
Body-centered cubic	BCC	3	1st	8	16000	4
	$\hbox {BCC}_2$		2nd	14	16000	3
Face-centered cubic	FCC	3	1st	12	32000	3
Graphene	Gra	2	1st	3	5000	6
Diamond	Dia	3	1st	4	8000	6
$\beta$-Sn	$\beta$-Sn	3	2nd	6	24000	4
Triangular	Tri	2	1st	6	6400	3
1-dimensional	1D	1	1st	2	10000	$\infty$
Simple square	Sq (SHC2)	2	1st	4	6400	4
	$\hbox {Sq}_2$		2nd	8	6400	3
Simple cubic	SC (SHC3)	3	1st	6	8000	4
Simple hyper-cubic	SHC4	4	1st	8	10000	4
	SHC5	5	1st	10	100000	4
	SHC6	6	1st	12	1000000	4

Figure 1 shows the simulation results for the simple square lattice within the first nearest neighbor (model Sq). At the initial state, all the sites are set to be A-sites, and one of them is changed to the O-site one by one at each (discrete) time step *t*. Thus ${N_\textrm{O}}$ is exactly proportional (equal) to *t* through the whole simulation process. The neighboring sites are altered into N-sites. At the very early stage ($t =$ 0–500), most of the O-sites are isolated from each other (i.e., only little overlap between their N-sites), resulting in a linear change in the numbers of the A-sites (${N_\textrm{A}}$) and the N-sites (${N_\textrm{N}}$). At the intermediate stage, the trends in ${N_\textrm{A}}$ and ${N_\textrm{N}}$ becomes nonlinear, because of overlap between the N-sites next to several O-sites. Eventually, sparsely remained A-sites are changed to O-sites one by one until the A-sites completely disappear at the final state $t_\textrm{final}$. Then the saturation fraction is obtained as the fraction of the O-sites at the final state; i.e., ${\rho _\textrm{sat}}= {\rho _\textrm{O}}(t_\textrm{final})$. The results for other cases are shown in Supplementary Discussion 1. The videos of random packing processes are available for the simple square lattice within the second nearest neighbor (model $\hbox {Sq}_2$) and the body-centered cubic lattice within the first nearest neighbor (model BCC) as Supplementary Video 1b and Supplementary Video 1c, respectively.

Figure 1d shows the saturation fractions ${\rho _\textrm{sat}}$ obtained by the random packing simulations for various lattice structures. It is found that, for all the lattice models, ${\rho _\textrm{sat}}$ is quite steady between four independent stochastic trials, and only slight deviations are observed. This result suggests that ${\rho _\textrm{sat}}$ is an intrinsic property of individual lattice structures. We also find a negative correlation between the saturation fraction ${\rho _\textrm{sat}}$ and the coordination number *D*, which is discussed in the subsequent sections.

### Mathematical model based on random graph

**Fig. 2:**
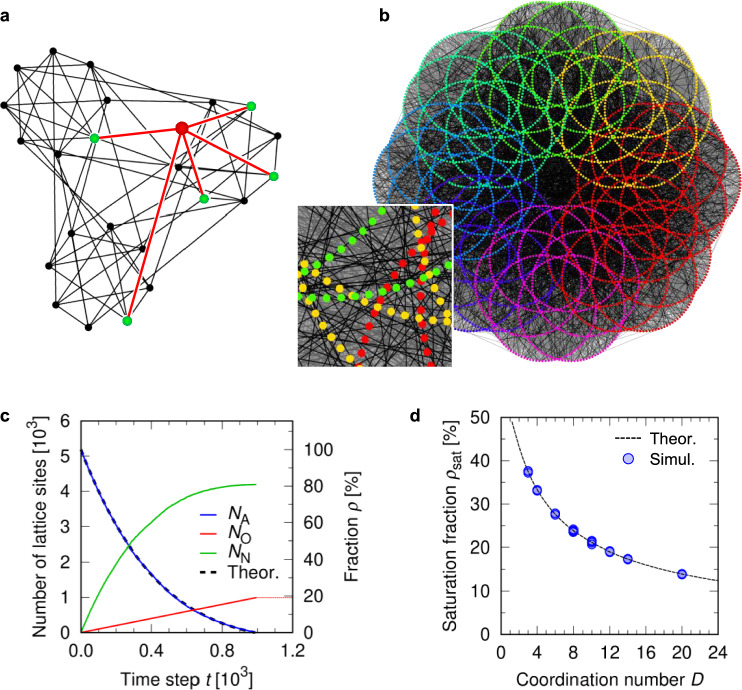
Random packing simulation for random regular graphs (RRGs). (**a**) Schematic of an RRG with 24 vertices of degree 5 (i.e., 5-regular). For visibility, a vertex and its adjacent vertices are highlighted in red and green, respectively. (**b**) An example of RRG simulation model (5148 vertices, 12-regular). The inset is an enlargement of a part of the model. The colored points represent vertices, and lines in gray or black represent edges in the graph, respectively. The color of the vertices indicates the subgraph to which the vertex belongs. The color of edges (gray to black) is varied for visibility. (**c**) Numbers of O-, A-, and N-sites as functions of time obtained by simulation for the above model (**b**). The dashed curve indicates the theoretical function of ${N_\textrm{A}}(t)$, i.e., Eq. (3), which is in good agreement with the simulation result (blue curve). (**d**) Relationship between saturation fraction ${\rho _\textrm{sat}}$ and coordination number *D* for all the RRG models, compared with theoretical prediction from Eq. (4).

In this section, we aim to develop a theoretical model of the random packing process. Since it is difficult to directly consider the complex character or order in specific lattice structures, we simplified the system as a random network structure instead of dealing with individual lattice structures. In addition, we assumed that the coordination number *D* is the major parameter to govern the saturation fraction ${\rho _\textrm{sat}}$. Such network system can be modeled as a random regular graph (RRG) ^35^. Here, the coordination number is defined as the number of the sites adjacent to an arbitrary site and thus is a characteristic of lattice or graph systems. Figure 2a shows a schematic illustration of an RRG. An RRG, where the coordination number of lattice sites is kept while their connection is shuffled, can be regarded as a random analogue of lattice structures. A vertex and an edge in a graph represent an atom site and a bond in a lattice, respectively. An example of random packing on a small RRG is shown in Supplementary Video 2. Below we establish a theoretical model to evaluate ${\rho _\textrm{sat}}$ for RRGs with a given coordination number *D*. The theoretical model for the random system can act as a basis of discussion for individual lattice structures.

The random packing process on lattices and RRGs is regarded as (discrete) time evolutions of ${N_\textrm{O}}$, ${N_\textrm{A}}$, and ${N_\textrm{N}}$. Then, the process is formulated as a simultaneous difference equation as follows:

1 $$\begin{aligned} \left\{ \begin{aligned} {N_\textrm{O}}(t+1)&= {N_\textrm{O}}(t) + 1, \\ {N_\textrm{A}}(t+1)&= {N_\textrm{A}}(t) - 1 - D \phi (t), \\ {N_\textrm{N}}(t+1)&= {N_\textrm{N}}(t) + D \phi (t). \end{aligned} \right. \end{aligned}$$

The function $\phi (t)$ represents the ratio of A-sites in the neighbors of the O-site newly selected at time *t*, which can be given in an analytical form by approximating the lattice as an RRG:

2 $$\begin{aligned} {\phi (t) = \frac{{N_\textrm{A}}(t) - 1}{({N_\textrm{A}}(t) - 1) + ({N_\textrm{N}}(t) - {N_\textrm{O}}(t))}.} \end{aligned}$$

This system of equations can be solved analytically, and we obtain ${N_\textrm{A}}(t)$ as

3 $$\begin{aligned} {N_\textrm{A}}(t)= & 1 - \frac{N-1}{D-2} \left( 1 - \frac{2t}{N-1} \right) + \frac{(N-1)(D-1)}{D-2} \left( 1 - \frac{2t}{N-1} \right) ^\frac{D}{2}. \end{aligned}$$

The detail of the derivation is explained in “Methods”. We rewrite this equation as a relationship between ${\rho _\textrm{A}}(t)$ and ${\rho _\textrm{O}}(t)$. Then ${\rho _\textrm{A}}$ for a sufficiently large system is given as

4 $$\begin{aligned} {\rho _\textrm{A}}({\rho _\textrm{O}})= & \frac{-1}{D-2}(1-2{\rho _\textrm{O}}) + \frac{D-1}{D-2} (1-2{\rho _\textrm{O}})^\frac{D}{2}. \end{aligned}$$

The saturation fraction ${\rho _\textrm{sat}}$ is given by ${\rho _\textrm{O}}$ satisfying the condition ${\rho _\textrm{A}}({\rho _\textrm{O}}) = 0$, where no more lattice site is available for dopant atoms. This equation can be analytically solved as follows:

5 $$\begin{aligned} {\rho _\textrm{sat}}(D) = \frac{1}{2} \left[ 1 - \left( \frac{1}{D-1} \right) ^\frac{1}{\frac{D}{2}-1} \right] . \end{aligned}$$

This is the principal result of the mathematical analysis in this study. This equation indicates that ${\rho _\textrm{sat}}$ is expressed as a simple function of the coordination number (i.e., the vertex degree of the RRG) *D*.

Although the exponent in Eq. (5) diverges at $D = 2$, ${\rho _\textrm{sat}}$ itself converges to a finite value:

6 $$\begin{aligned} \lim _{D \rightarrow 2} {\rho _\textrm{sat}}(D) = \frac{1-e^{-2}}{2} \approx 0.432. \end{aligned}$$

Note that the case of $D=2$ corresponds to the one-dimensional lattice, and this result is revisited in Discussion.

### Numerical simulation of random packing on random graph

For validation of the theoretical solution derived in the previous section, we performed a series of random packing simulation with RRGs. Fig. 2b shows an example of RRG simulation model. The details of the method to generate RRG models are described in Methods and Supplementary Methods 1. We developed 30 RRG models with the vertex degree (coordination number) *D* varied from 3 to 20 (see Table 2).

**Table 2:** Examined random regular graphs.

Coordination number (degree) *D*	Number of models
3	3
4	3
6	3
8	5
10	5
12	4
14	3
20	4

Figure 2c shows the simulation result for a 12-regular graph model, where each vertex has twelve adjacent vertices. The complete simulation data for all RRGs are available in Supplementary Discussion 2. We find excellent agreement in the ${N_\textrm{A}}$-*t* between the theoretical model and simulation results for RRGs. Figure 2d shows ${\rho _\textrm{sat}}$ as a function of *D* for all the RRG models compared with the theoretical estimation (Eq. 5), which also shows good accordance in theoretical prediction and simulation results. It is remarkable that there is no fitting parameters in the theoretical model. This result indicates that the theoretical model can properly describe the random packing process on graphs, i.e., networks with adjacency defined. Thus, we can discuss the trend in ${\rho _\textrm{sat}}$ for lattices (Fig. 1d) on the theoretical basis.

## Discussion

**Fig. 3:**
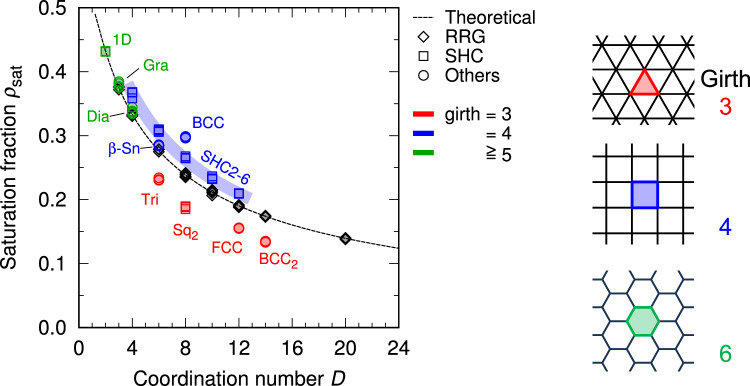
Relationship between coordination number *D* and saturation fraction ${\rho _\textrm{sat}}$ evaluated by simulation and theoretical analysis. Right schematics exemplify girth of typical lattices: Intuitively, a lattice with *n*-membered ring has girth *n*.

Figure 3 shows the saturation fractions ${\rho _\textrm{sat}}$ as a function of the coordination number *D* evaluated by the numerical simulations and the theoretical model. It is by no means trivial that ${\rho _\textrm{sat}}$ for many crystal lattice structures can be approximated by the theoretical model based on the random graph. Our formulation successfully captures the universal trend underlying all real and imaginary lattices.

Here we focus on the discrepancy in ${\rho _\textrm{sat}}$ between simulation for lattice models and theoretical analysis. In some cases, the deviation from the theoretical prediction is relatively large. For example, the saturation fractions of the series of simple hypercubic lattices (including the simple square and simple cubic lattices) exceed the theoretical prediction, and the trend is quite steady. Meanwhile, the BCC lattice exhibits a more complex trend according to the definition of adjacency: The case within 1NN results in an excessive saturation fraction (i.e., above the theoretical curve in Fig. 3), while the saturation fraction of $\hbox {BCC}_2$ model (adjacent within 2NN) is deficient (i.e., below the theoretical curve).

**Fig. 4:**
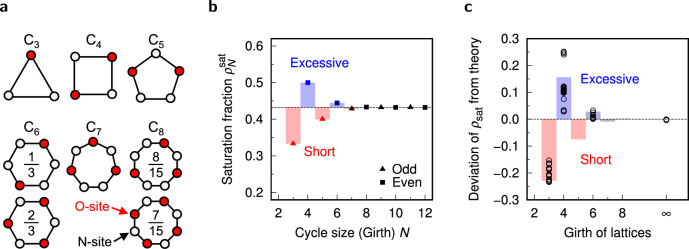
Comparison between trend in cycle graphs and the simulation result for lattices. (**a**) Small cycle graphs $\hbox {C}_N$ ($N = 3$–8) at saturation. The O-sites are colored in red. In the cases of $N =$ 3, 4, 5, and 7, the saturated configuration is uniquely determined, while several configurations are possible as the saturated state for $N =$ 6, 8, and larger numbers. The fractions in the graphs for $N =$ 6 and 8 indicate the probabilities of the corresponding configurations (or its equivalences) being realized. (**b**) Saturation fraction of cycle graphs as a function of cycle size (girth) *N*. The values are indicated by bars and points for visibility. The dashed line represents ${\rho _\textrm{sat}}(2)$, which is corresponding to the case of $N \rightarrow \infty$. (**c**) Deviation of saturation fraction for lattice models from theoretical prediction as a function of girth. The vertical axis is given in the form of “error”, i.e., $\frac{\rho ^\textrm{lattice}_\textrm{sat}}{\rho ^\textrm{theory}_\textrm{sat}}-1$. The colored bars indicate the error for cycle graphs, which is directly converted from (**b**) by normalizing with ${\rho _\textrm{sat}}(2)$. The points at girth $\rightarrow \infty$ are corresponding to the 1D lattice model.

Interestingly, the origin of these deviations can be qualitatively explained to some extent through graph-theoretical discussion. We introduce a graph invariant called girth, which is defined as the length (the number of edges) in the shortest cycle in the graph. The data points in Fig. 3 are colored by girth for each lattice structure. It is clearly found that the cases of shortage and excess in ${\rho _\textrm{sat}}$ correspond to girth = 3 and 4, respectively. The lattices with a larger girth (e.g., diamond structure, having girth 6) have a relatively small deviation from the theoretical prediction. The relationship between girth and the deviation of ${\rho _\textrm{sat}}$ is explained by the trend in ${\rho _\textrm{sat}}$ of finite cycle graphs (Fig. 4a). Here let ${\rho ^\textrm{sat}_N}$ be the saturation fraction of the cycle graph with *N* vertices (equivalently, girth = *N*). Then, the exact solution of ${\rho ^\textrm{sat}_N}$ is given by the following recurrence relation:

7 $$\begin{aligned} {\rho ^\textrm{sat}_N}= & \frac{1+X_{N-3}}{N}, \end{aligned}$$

8 $$\begin{aligned} \left\{ \begin{aligned} X_0&= 0, \\ X_1&= 1, \\ X_N&= \left( 1- \frac{1}{N} \right) X_{N-1} + \frac{2}{N} X_{N-2} + \frac{1}{N}, \end{aligned} \right. \end{aligned}$$

where the sequence $X_n$ $(n=1,..., N-3)$ is related to a property of subgraphs of the cycle graph $\hbox {C}_N$. The detailed derivation is provided in Supplementary Discussion 3. Figure 4b shows ${\rho ^\textrm{sat}_N}$ up to $N = 12$. It is numerically confirmed that ${\rho ^\textrm{sat}_N}$ rapidly converges to the theoretical value calculated for the 1D lattice, i.e., $(1-e^{-2})/2 \approx 0.432$ (see Eq. (6)), accompanied with a damped oscillation-like behavior. If *N* is an odd number, ${\rho ^\textrm{sat}_N}$ is smaller than the theoretical value; otherwise, if *N* is even, ${\rho ^\textrm{sat}_N}$ is larger. This tendency is comparable to the relationship between girth and the deviation of ${\rho _\textrm{sat}}$ in the lattice models from the theoretical values (Fig. 4c). In the case of the lattice structures with girth 3, the deviation tends to be smaller than the theoretical value for RRGs, while lattices of girth 4 enable denser packing.

From the definition, a lattice with girth *n* includes *n*-cycles as a subgraph, and no smaller cycles are included. For example, the girth of the face-centered cubic (FCC) lattice is 3, and the FCC lattice includes many 3-cycles (Each lattice site belongs to 24 distinct 3-cycles). Then, the restrictions on 3-cycles applies to the FCC lattice as well, and therefore only one O-site can exist in each of those cycles at maximum. Such restriction acts to decrease ${\rho _\textrm{sat}}$ of the lattice, as seen in Fig. 4c. Even though the FCC lattice includes 4-cycles and larger cycles, the effect of the 3-cycles is expected to be dominant, as is inferred from the analysis for cycle graphs (Fig. 4b). On the other hand, the girth of the BCC lattice is 4. From Fig. 4b, The cycles with small even girth is likely to enable more efficient (denser) packing than the cases of random networks or larger cycles. The lattice structures with girth 6 (diamond, graphene) have relatively small deviations from theoretical prediction, presumably because a 6-cycle is not so effective to cause a large deviation, as is seen in Fig. 4b. Although RRGs may have small girth, the effect of small cycles is expected to be marginal because the number of such small cycles is negligible and unlikely to make a substantial influence on the properties of the entire graph, unlike the cases of uniform and highly ordered lattice structures.

Except for the $\beta$-Sn structure, the examined structures with an even girth have only “even cycles”: Such graphs are called bipartite ^36^. In the case of bipartite graphs, the densest packing can be realized in principle by setting O-sites and N-sites alternately. Locally ordered structure is actually observed in the saturated configuration of the simple square lattice (Fig. 1). This situation is similar to the geometrical frustration of spins (magnetic moment) in certain antiferromagnetic materials ^37,38^: The order of spin in a BCC antiferromagnet (e.g., Cr) can be collinear because of the bipartite nature of the BCC lattice, while a FCC antiferromagnet (e.g., Fe-Mn) should have a more complex order ^39^. A collinear antiferromagnetic order is impossible in a non-bipartite lattice.

In summary, the saturation fraction ${\rho _\textrm{sat}}$, a characteristic property of lattices, can be approximated as a function of the coordination number (or vertex degree) by theoretical model based on random graphs. The basic trend in ${\rho _\textrm{sat}}$ over typical lattices is in good agreement with the theoretical model prediction. Even the deviation in ${\rho _\textrm{sat}}$ from the theoretical prediction can be qualitatively explained by considering another graph property, girth. Note that both the coordination number and girth of a lattice are local properties, which can be easily obtained once the lattice structure and adjacency are defined.

**Fig. 5:**
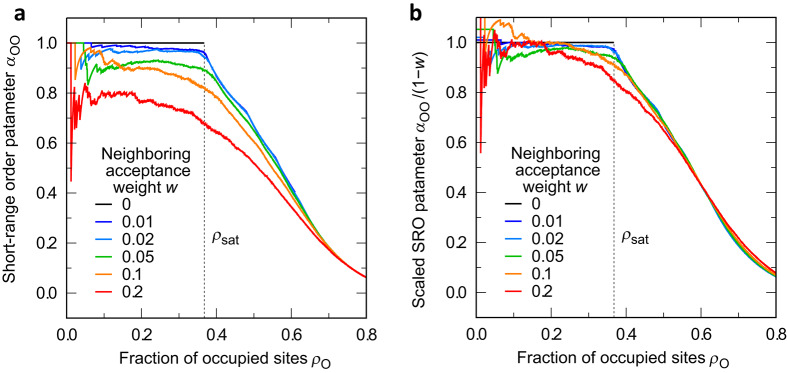
Warren–Cowley short-range order parameter for dopant-dopant (O–O) pair as a function of O-site fraction. (**a**) As calculated; (**b**) rescaled by dilute limit (${\alpha _\textrm{OO}}\rightarrow 1-w$ for ${\rho _\textrm{O}}\rightarrow 0$).

It should also be noted that dopants in real materials can be neighbored, even if they strongly repel each other, because of the finiteness of the repulsive energy. To address this issue, we extended the simulation model with incomplete repulsion by introducing a new parameter, *w*, to control the probability of accepting neighboring of repelling dopants, which is related to the Boltzmann factor. Details of the simulation model and the simulation results are expressed in Methods and Supplementary Discussion 4. In this case, the Warren-Cowley SRO parameter for the O-O pair (i.e., the dopant-dopant pair), ${\alpha _\textrm{OO}}$, is varied according to the strength of repulsion, while the case of complete repulsion always results in ${\alpha _\textrm{OO}}= 1$. Thus, it is interesting to investigate the change in the SRO parameter with increasing dopant fraction, $\rho _\textrm{O}$. Figure 5 shows simulation results of the Warren-Cowley parameter ${\alpha _\textrm{OO}}$ as a function of $\rho _\textrm{O}$ for the Sq lattice. It is remarkable that the trend in the SRO parameter clearly changes at the saturation fraction, ${\rho _\textrm{sat}}$, in all the cases. Thus, the saturation fraction is found to be an intrinsic characteristic of lattice system indicating a sort of transition (or crossover) of the states of SRO.

Next, we apply those results to discussion for the actual materials. The present discussion can be extended in a straightforward way to multi-principal-element alloys by regarding each constituent as a dopant element. Then, an interesting insight arises for high-entropy alloys (HEAs) and medium-entropy alloys (MEAs): Usually, an alloy with four or more elements is called HEA, while a three-element alloy is called MEA *just by convention* rather than in a physical sense. Focusing on the BCC lattice as an example, the SROs of ternary and quaternary alloys are expected to be qualitatively different. Supposing equiatomic compositions, the fractions of each element are 25 % and 33 % in a quaternary alloy (HEA) and a ternary alloy (MEA), respectively, and those values are below and above the saturation fraction of the BCC lattice (${\rho _\textrm{sat}}\approx 30 \%$). Thus, even if there are dopant atoms repelling each other in MEA, they are statistically inevitable to be neighboring with the same species. This situation is essentially different from the case of an HEA with four or more elements, where the repulsive elements can be kept non-neighboring because of the atom fraction lower than ${\rho _\textrm{sat}}$. In addition, as is seen in Fig. 5, the trend in the SRO parameter substantially changes below and above ${\rho _\textrm{sat}}$, and the system undergoes a sort of crossover at that point. Hence, we can give a new viewpoint to distinguish HEAs and MEAs in such a context via the saturation fraction. Meanwhile, the FCC lattice requires at least six elements to satisfy the non-neighboring preference (since ${\rho _\textrm{sat}}\approx 16 \%$ for the FCC lattice). Those features can be adjusted by changing the compositions of each element. Such point of view can provide another degree of freedom for alloy design.

Moreover, the present analyses can be extended to the hydrogen-metal systems, where hydrogen atoms are stored in metal as protons or molecules ^40,41^. There we consider a lattice of interstitial sites to be occupied by hydrogen atoms (protons), instead of the explicit lattice of metal atoms. Since hydrogen atoms in metals repel each other by the Coulomb interaction ^42,43^, the situation is very close to the present discussion. Even if the interstitial lattice is not very simple, a rough estimation can be made for the capacity of hydrogens under sufficiently low external pressure as ${\rho _\textrm{sat}}$, for which we only need several simple lattice properties (i.e., coordination number and girth) to be accessible.

Another potential application is for metallic glasses or amorphous metals, which also have been attracting attention for their excellent features ^44,45^. Thanks to generalization and universality of the mathematical model, that is applicable for non-crystalline systems, and we can even estimate ${\rho _\textrm{sat}}$ quantitatively to some extent if just the coordination number and girth are available. It should be noted that this cannot be achieved without theoretical discussion.

## Conclusion

It is not a trivial problem how much dopant atoms can exist in a lattice if they are placed at random under the non-neighboring condition. We addressed this problem by comprehensive numerical simulations and theoretical modeling to evaluate the fraction of the lattice sites occupied by dopant atoms, especially at the saturated state. We developed a theoretical model based on random graphs, with which we revealed the basic trend in the saturation fraction of repulsive dopants as a function of the coordination number. Further analyses of subsystems of lattices indicated that another graph property, namely girth, makes a considerable and systematic effect on the saturation fraction. The discrepancy in the saturation fractions between the simulation results and the theoretical prediction was explained well by considering the effect of girth. Those results provide a generalized insight that the amount of dopant dissoluble in an alloy system is determined by several simple graph properties of the lattice structure. This conclusion is not limited to certain specific materials or structures but potentially applicable for universal material systems, such as multi-principal element alloys.

## Methods

### Random packing simulation of lattice models

We examined 14 cases with different 12 lattice structures as follows; body-centered cubic (BCC), face-centered cubic (FCC), diamond (Dia), $\beta$-Sn, graphene (Gra), triangular (Tri), one-dimensional (1D), simple square (Sq), simple cubic (SC), and 4–6-dimensional simple hyper-cubic (SHC) lattices. Note that the 1D, Sq, and SC lattices can also be regarded as low dimensional cases in the SHC lattice class. The details of the examined models are shown in Table 1. Not only typical lattice structures such as the BCC or FCC lattices, but also several imaginary lattice structures (e.g., simple hyper-cubic lattices) were examined for the purpose of taking a general perspective on arbitrary lattices.

For the BCC and Sq structures, we considered up to the second nearest neighbor (2NN) sites as adjacent sites, in addition to the case with only the first nearest neighbors (1NN). Note that the character of lattice graphs depends on the definition of adjacency (i.e., up to what nearest neighbors are considered as adjacent). Hence, the properties of the graphs with and without the 2NN sites are substantially different, and those cases should be treated as separate simulation cases. Basically, we regard the 1NN sites as adjacent. Since the 1NN of the $\beta$-Sn lattice is equivalent to that of the diamond lattice, we only examined the case within the 2NN for the $\beta$-Sn lattice. It should be noted that the terms of “neighboring” and “adjacent” mean the vicinity in the geometrical and graph-theoretical senses, respectively.

The periodic boundary condition was imposed on all the simulation models according to the dimension of the systems to avoid the effect of margin regions. Simulation cell size, i.e., the number of the lattice sites (vertices) *N*, was set to be sufficiently large. Four independent stochastic trials were conducted for all the cases.

### Extension of simulation model for incomplete repulsion

**Fig. 6:**
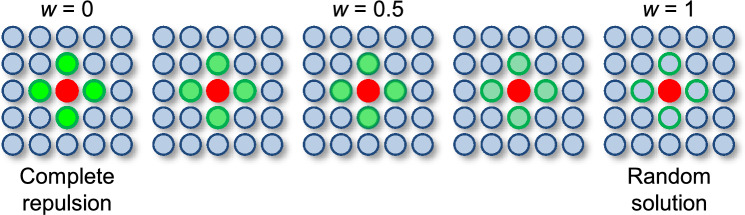
Schematic of the extended model. The weight parameter *w* determines the relative probability of selecting an N-site: For $w = 0$, the model is equivalent to the original model (i.e., complete repulsion), while the case of $w = 1$ mimics a random solid solution.

Here we extend the simulation model with a non-zero probability to allow dopants adjacent to each other (hereafter referred to as the “extended model”) from the “original model”, where dopants are placed in the exactly non-neighboring manner. The concept of the extended model is shown in Fig. 6.

To allow dopants adjacent to each other, we introduced the acceptance weight parameter, *w*, to the simulation model, which represents the relative probability that a neighboring site (N-site) is selected as a dopant site (i.e., O-site). If we set $w = 0$, an N-site has no chance to be selected as the O-site; this case is exactly equal to the original model. If we set $w = 1$, an N-site has as much chance as an A-site, and there is no difference between the N- and A-sites; this situation is equivalent to the random solid solution case. An intermediate weight, $0<w<1$, can realize the situation of a finite possibility of neighboring of the repulsive pair. Thus, the extended model can consistently bridge the complete non-neighboring case (the original model) and the random solid solution. If a site is adjacent to *n* dopants, the weight of that site is set to $w^n$.

### Derivation of time-evolution equation and solution

Below, we solve the difference equation (1).

Firstly we derive the function form of Eq. (2). There it is needed to consider a feature of RRGs, namely the regularity (the equality in the degree of all vertices). Considering the regularity, it is found that an N-site is less likely to be adjacent to the new O-site than an A-site is, because at least one bond at an N-site is known to be adjacent to another (already existing) O-site. After all, the probabilities for the newly selected O-site being adjacent to an A-/N-site are proportional to the total numbers of the bonds at A-/N-sites whose destination is not an existing O-site. For the A-site, the number such bonds is easily counted as ${B_\textrm{A}}= D({N_\textrm{A}}- 1)$ because an A-site is not adjacent to an O-site. Here reduction by 1 is due to one site selected from set of A-sites to be the new O-site. For the N-site, the number of candidate bonds are calculated as ${B_\textrm{N}}= D{N_\textrm{N}}- D{N_\textrm{O}}$, where the second term represents the number of bonds incident to the existing O-sites. Then, the probability for the new O-site being adjacent to an A-site is given as

9 $$\begin{aligned} \phi (t) = \frac{{B_\textrm{A}}}{{B_\textrm{A}}+{B_\textrm{N}}}, \end{aligned}$$

from which we directly obtain Eq. (2).

Since all the sites are A-site at the initial state, the initial condition is given as

10 $$\begin{aligned} \left\{ \begin{aligned} {N_\textrm{O}}(0)&= 0, \\ {N_\textrm{A}}(0)&= N, \\ {N_\textrm{N}}(0)&= 0. \end{aligned} \right. \end{aligned}$$

The total number of the lattice sites *N* is unchanged through the process, and thus the following relationship is satisfied at each time *t*;

11 $$\begin{aligned} {N_\textrm{O}}(t) + {N_\textrm{A}}(t) + {N_\textrm{N}}(t) = N. \end{aligned}$$

Then, we can directly derive ${N_\textrm{O}}(t) = t$ from the time evolution equation Eq. (1) and the initial condition Eq. (10). In addition, ${N_\textrm{N}}(t)$ is also obtained as follows:

12 $$\begin{aligned} {N_\textrm{N}}(t) = N - t - {N_\textrm{A}}(t), \end{aligned}$$

and thus

13 $$\begin{aligned} \phi (t) = \frac{{N_\textrm{A}}(t) - 1}{N - 1 - 2t}. \end{aligned}$$

Eventually, we only have to solve the explicit form of ${N_\textrm{A}}(t)$ to solve the system of equations. From the obtained relations, the equation of ${N_\textrm{A}}(t)$ is modified to the following form:

14 $$\begin{aligned} {N_\textrm{A}}(t+1) = {N_\textrm{A}}(t) - 1 - D \cdot \frac{{N_\textrm{A}}(t) - 1}{N - 1 - 2t}. \end{aligned}$$

Here we replace difference (${N_\textrm{A}}(t+1) - {N_\textrm{A}}(t)$) in the equation with differentiation ($(d{N_\textrm{A}}/dt) {\Delta t}$, where ${\Delta t}=1$):

15 $$\begin{aligned} & \frac{d{N_\textrm{A}}(t)}{dt} = - 1 - D \cdot \frac{{N_\textrm{A}}(t) - 1}{N - 1 - 2t}, \nonumber \\ \therefore & \frac{d{N_\textrm{A}}(t)}{dt} + \frac{D}{N-1-2t} {N_\textrm{A}}(t) = -1 + \frac{D}{N-1-2t}. \end{aligned}$$

This equation is a first-order linear ordinary differential equation for ${N_\textrm{A}}(t)$ and known to be analytically solved. We obtain the solution as Eq. (3).

Next, Eq. (3) is rewritten into a relationship between ${\rho _\textrm{A}}$ and ${\rho _\textrm{O}}$. The both sides of Eq. (3) are divided by *N*:

16 $$\begin{aligned} \frac{{N_\textrm{A}}(t)}{N}= & \frac{1}{N} - \frac{1}{D-2} \left( 1- \frac{1}{N} \right) \left( 1 - \frac{2t}{N-1} \right) \nonumber \\ & + \frac{D-1}{D-2} \left( 1- \frac{1}{N} \right) \left( 1 - \frac{2t}{N-1} \right) ^\frac{D}{2}. \end{aligned}$$

By taking the limit of $N \rightarrow \infty$, we obtain Eq. (4). Note that ${N_\textrm{A}}/N \rightarrow {\rho _\textrm{A}}$ and $t/N \rightarrow {\rho _\textrm{O}}$ at the limit.

### Generation of regular random graph

Generating an RRG itself is not trivial, and the basic generation methods are sometimes accompanied by trial-and-error procedures ^46,47^. Since it is difficult to make an RRG with a large number of vertices and edges in a fully random way (with the uniform probability distribution), we employed a brief way to make graph models, with which we can generate a nearly random graph from smaller regular subgraphs. We developed an algorithm to generate an RRG with *mn* vertices of degree $c+d$ from one RRG with *m* vertices of degree *c* and *m* RRGs with *n* vertices of degree *d*. Subgraphs are connected by additional edges under a certain rule. As large an RRG as necessary can be complied by recursively applying this method with a proper series of *c*, *d*, *m*, and *n*. The detailed explanation of the algorithm is shown in Supplementary Methods 1.

For example, the simulation model in Fig. 2 has 5184 vertices of degree 12 (i.e., $N = 5184$ and $D = 12$). This entire graph is compiled from eight subgraphs, which are also regular graphs having 648 vertices of degree 5 each. Each of these subgraphs is also compiled from six smaller subgraphs which are null graphs having 108 vertices. Here, a null graph is a graph without edges. By introducing such a hierarchical structure, simple recursive construction of an RRG is enabled without any trial-and-error process.

In this study, we fixed the number of hierarchical layers to one or two (i.e., one or two recursive cycles), and started with the null graphs (i.e., $d=0$) at the lowest level of hierarchy. We also fixed several parameters as $N = 5184$ and $c = m-1$. Then we picked up all the possible combinations of *c*, *d*, *m*, and *n* that satisfy the above conditions for each degree (coordination number) *D*, and generated the RRG simulation models for each combination. As a result, we created 3–5 RRG models for each *D* and conducted one stochastic trial for each model. The detail of the generated models are shown in Supplementary Methods 2.

## Supplementary Information

Supplementary Information 1.

Supplementary Information 2.

Supplementary Information 3.

Supplementary Information 4.

Supplementary Information 5.

Supplementary Information 6.

## Data Availability

The authors declare that all the data supporting the findings in this study are available within the article and/or Supplementary Information. All other data are available from the corresponding author upon request.

## Code availability

The codes for random graph generation and random packing simulation are developed by the authors and available from the corresponding author upon request.
