# Supplementary material for: Graph-theoretic analyses of saturation fraction of repulsive dopants in solid solutions
Source: Sci Rep. 2026 Mar 12;16:7650. doi: 10.1038/s41598-025-30829-1 (PMC12982781; doi:10.1038/s41598-025-30829-1)
Supplement: Supplementary file 1 — Supplementary Information 1. [file 41598_2025_30829_MOESM1_ESM.pdf]

# Supplementary Information for
# Graph-Theoretic Analyses of Saturation Fraction of Repulsive Dopants in Solid Solutions

Atsushi Kubo[1*] and Yosuke Abe[1]

[1] Japan Atomic Energy Agency, 2-4 Shirakata, Tokai-mura, 319-1195, Ibaraki, Japan .

*Corresponding author(s). E-mail(s): kubo.atsushi@jaea.go.jp;

## Supplementary Videos

### Video1a_Simulation_Sq

Random packing simulation of simple square lattice within the first nearest neighbor (Sq).

### Video1b_Simulation_Sq2

Random packing simulation of simple square lattice within the second nearest neighbor ($Sq_2$).

### Video1c_Simulation_BCC

Random packing simulation of BCC lattice within the first nearest neighbor (BCC).

### Video2_Simulation_RRG_example

Schematic (example) of random packing process for a small random regular graph.

### Video3_Simulation_RRG12

Random packing simulation of a random regular graph model (12-regular).

# Supplementary Methods 1: Algorithm for Generating Random Regular Graph Model

Here we explain the method to make a random regular graph (RRG) by compiling smaller subgraphs. Supplementary Figure 1 schematically shows the algorithm.

At the beginning, we prepare one RRG with $m$ vertices of degree $c$ and $m$ RRGs with $n$ vertices of degree $d$; hereafter those graphs are denoted by $G$ and $g_i$ ($i = 1, ..., m$), respectively. A RRG with $mn$ vertices of degree $c + d$ is to be created from $G$ and $\{g_i\}$. Each vertex $i$ in $G$ is related to $g_i$ in a one-to-one manner, and an edge between $i$ and $j$ in $G$ can be identified to the pair of $g_i$ and $g_j$.

1. We obtain a graph disjoint union of $\{g_i\}$, which is denoted by $\mathit{\Gamma} := \oplus_i g_i$ (We identify $g_i$ as the corresponding subgraph in $\mathit{\Gamma}$). Obviously $\mathit{\Gamma}$ has $mn$ vertices of degree $d$. Each of $g_i$ is disconnected from each other at this stage (see the left panel in Supplementary Fig. 1).
2. For each edge (i.e., $i$-$j$ pair) in $G$, we give a random bijection from $g_i$ to $g_j$. In other words, a one-to-one correspondence is assigned between the vertices in $g_i$ and $g_j$ at random.
3. The above one-to-one correspondence between subgraphs are used for definition of additional edges and newly included to the edge set of $\mathit{\Gamma}$ (see the right panel in Supplementary Fig. 1).

It is easily found that $\mathit{\Gamma}$ is a simple graph and especially a $(c + d)$-regular graph. From the definition of $g_i$, an arbitrary vertex in $g_i$ should be adjacent with exactly $d$ vertices in $g_i$ (via colored edges in Supplementary Fig. 1). Each of $g_i$ is connected exactly $c$ other subgraphs $g_j$ ($j \neq i$), and those connections are interpreted as bijections between $g_i$ and $g_j$. Thus, a vertex in $g_i$ is connected to one vertex in each of $g_j$ (via gray edges in Supplementary Fig. 1), and then we find that the degree of any vertex is $c + d$. Note that graph $G$ is used for determining the connection between subgraphs $\{g_i\}$. In this study, we set $G$ to be a complete graph, where any pair of vertices are adjacent, and thus $c = m - 1$.

As explained in Methods, this algorithm can be applied recursively to get a huge RRG. The present simulation models were generated by repeating this cycle once or twice, started with null graphs. The construction precess is characterized by the set of the parameters $m, n, c$, and $d$. When it is applied recursively, as many sets of the parameters as the number of hierarchical layers (cycles) are required.

This algorithm does not include any trial-and-error process and thus is useful for the present purpose to create a nearly random graph, although not exactly random.

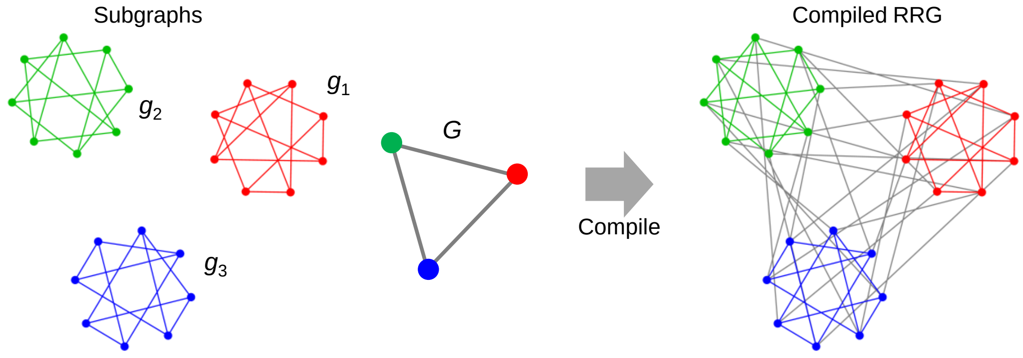


**Supplementary Figure 1** Schematic illustration of a random regular graph with 24 vertices of degree 5. Since this example is 5-regular, all the vertices have exactly five incident edges. To make the whole RRG, firstly we make a graph union of subgraphs, which is a simple collection of subgraphs (left). In this example, there are three constituent subgraphs $g_1$, $g_2$, and $g_3$, indicated in red, green, and blue, respectively. Those subgraphs are 3-regular, and thus each vertices are adjacent with other three vertices in the same subgraph. Secondly, we combine subgraphs by a random bijection, which defines adjacency (edges) between two different subgraphs (indicated as gray lines). Then, any vertex has five incident edges; i.e., three edges included in the original subgraph and two additional edges connected to other subgraphs.

# Supplementary Methods 2: Examined Random Graph Models

Based on the algorithm shown in Supplementary Methods 1, we created simulation models by determining the parameter set ($m$, $n$, $c$, $d$).

To make a RRG with a given number of vertices ($N$) and a given degree ($D$), The possible combinations of the parameter set are obtained as (positive) integer solutions of an algebraic equation.

In the case with one hierarchical layer, the equation is as follows:

$$\begin{cases} N = m_1 n_1, \\ D = c_1 + d_1. \end{cases} \tag{S1}$$

Any combination of ($m_1$, $n_1$, $c_1$, $d_1$) can be a source to generate a RRG with $N$ vertices of degree $D$. Furthermore, we imposed additional conditions for simplicity: $d_1 = 0$, $c_1 = m_1 - 1$. The former equation means that all of $g_i^1$ are null graphs (i.e., set of unconnected vertices), and the latter one that $G^1$ is a complete graph, respectively. Then the solution of the equation is, if any, uniquely determined: $m_1 = D + 1$ and $n_1 = N/(D + 1)$. Note that $m_1$ and $n_1$ are integers, and thus the equation does not necessarily has a solution.

In the case with two hierarchical layers, the equation is as follows:

$$\begin{cases} N = m_1 n_1, \\ D = c_1 + d_1. \\ n_1 = m_2 n_2, \\ d_1 = c_2 + d_2. \end{cases} \tag{S2}$$

Also in this case, we imposed additional conditions in a similar way: $d_2 = 0$, $c_1 = m_1 - 1$, $c_2 = m_2 - 1$. It is possible that there are more than one solutions. We collected all the solutions and generated RRGs corresponding to each solution. The resultant parameter sets are listed in Supplementary Table 1.

**Supplementary Table 1** Examined random regular graphs, represented by the corresponding solutions of Eqs. (S1) and (S2).

| Whole model | Layer 1 | | Layer 2 | |
|---|---|---|---|---|
| | $G^1$ | $g_i^1$ | $G^2$ | $g_i^2$ |
| $(N, D)$ | $(m_1, c_1)$ | $(n_1, d_1)$ | $(m_2, c_2)$ | $(n_2, d_2)$ |
| (5184, 3) | (4, 3) | (1296, 0) | - | - |
| | (2, 1) | (2592, 2) | (3, 2) | (864, 0) |
| | (3, 2) | (1728, 1) | (2, 1) | (864, 0) |
| (5184, 4) | (2, 1) | (2592, 3) | (4, 3) | (648, 0) |
| | (3, 2) | (1728, 2) | (3, 2) | (576, 0) |
| | (4, 3) | (1296, 1) | (2, 1) | (648, 0) |
| (5184, 6) | (2, 1) | (2592, 5) | (6, 5) | (432, 0) |
| | (4, 3) | (1296, 3) | (4, 3) | (324, 0) |
| | (6, 5) | (864, 1) | (2, 1) | (432, 0) |
| (5184, 8) | (9, 8) | (576, 0) | - | - |
| | (2, 1) | (2592, 7) | (8, 7) | (324, 0) |
| | (4, 3) | (1296, 5) | (6, 5) | (216, 0) |
| | (6, 5) | (864, 3) | (4, 3) | (216, 0) |
| | (8, 7) | (648, 1) | (2, 1) | (324, 0) |
| (5184, 10) | (3, 2) | (1728, 8) | (9, 8) | (192, 0) |
| | (4, 3) | (1296, 7) | (8, 7) | (162, 0) |
| | (6, 5) | (864, 5) | (6, 5) | (144, 0) |
| | (8, 7) | (648, 3) | (4, 3) | (162, 0) |
| | (9, 8) | (576, 2) | (3, 2) | (192, 0) |
| (5184, 12) | (2, 1) | (2592, 11) | (12, 11) | (216, 0) |
| | (6, 5) | (864, 7) | (8, 7) | (108, 0) |
| | (8, 7) | (648, 5) | (6, 5) | (108, 0) |
| | (12, 11) | (432, 1) | (2, 1) | (216, 0) |
| (5184, 14) | (4, 3) | (1296, 11) | (12, 11) | (108, 0) |
| | (8, 7) | (648, 7) | (8, 7) | (81, 0) |
| | (12, 11) | (432, 3) | (4, 3) | (108, 0) |
| (5184, 20) | (4, 3) | (1296, 17) | (18, 17) | (72, 0) |
| | (6, 5) | (864, 15) | (16, 15) | (54, 0) |
| | (16, 15) | (324, 5) | (6, 5) | (54, 0) |
| | (18, 17) | (288, 3) | (4, 3) | (72, 0) |

# Supplementary Discussion 1: Random Packing Simulation Results on Lattice Models

Supplementary Figure 2 shows the temporal evolutions of $N_{\mathrm{O}}$, $N_{\mathrm{A}}$, and $N_{\mathrm{N}}$ obtained for each lattice structure model. While we conducted four trials for each model, we only showed one of them because the results in four trials were very close to each other.

# Supplementary Discussion 2: Random Packing Simulation Results on Random Graph Models

Supplementary Figure 3 shows the temporal evolutions of $N_{\mathrm{O}}$, $N_{\mathrm{A}}$, and $N_{\mathrm{N}}$ obtained for RRG models. One case for each degree $D$ is shown. No clear difference is observed between the models with same $D$.

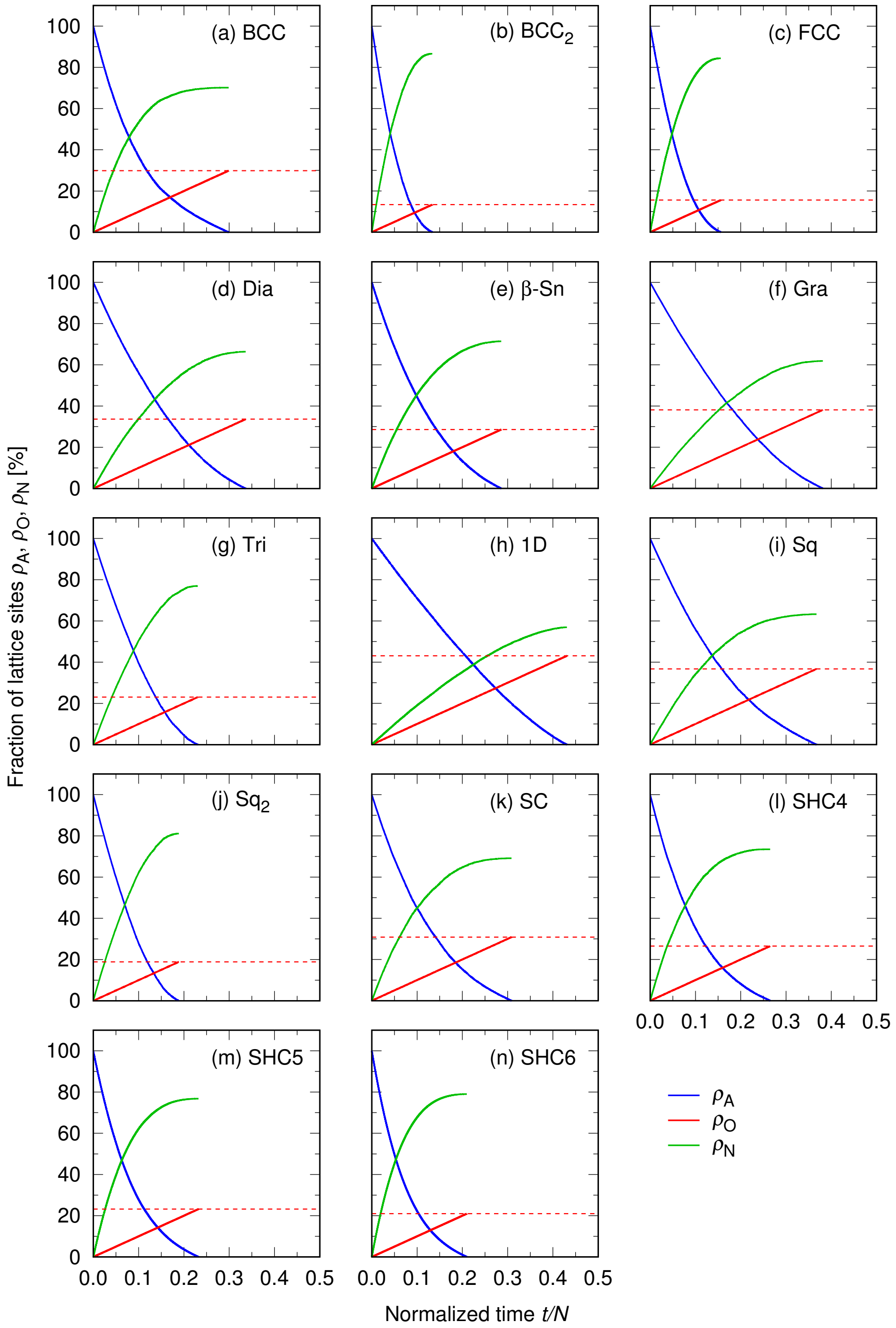


**Supplementary Figure 2** Temporal evolution of fractions of O-, A-, and N-sites for lattice models. The horizontal dashed red lines indicate the saturation fraction $\rho_{\mathrm{sat}}$.

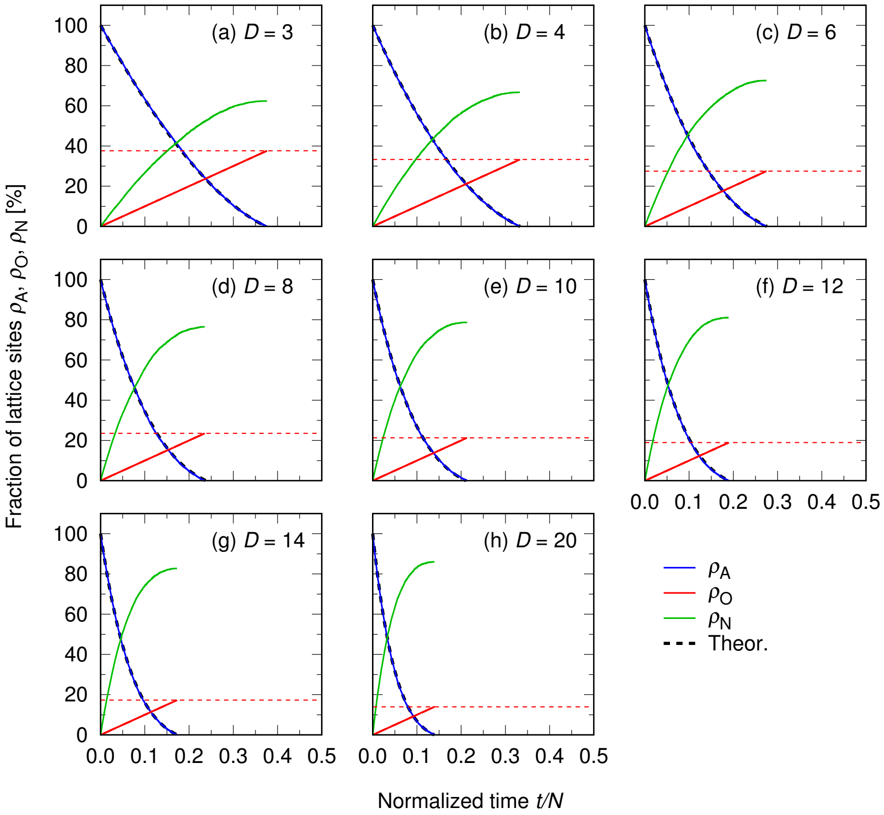


**Supplementary Figure 3** Temporal evolution of fractions of O-, A-, and N-sites for RRG models. The horizontal dashed red lines indicate the saturation fraction $\rho_{\text{sat}}$. The black dashed curves indicate the theoretical function of $N_{\text{A}}(t)$, i.e., Eq. (3), normalized by $N$.

# Supplementary Discussion 3: Derivation of Saturation Fraction for Finite Cycle Graphs

Unlike the cases of lattices and RRGs, the saturation function of finite cycle graphs can be exactly solved in a combinatorial way. Supplementary Figure 4 shows a schematic illustration of derivation of the saturation fraction for $N$-cycle, $\rho_N^{\rm sat}$. For derivation, we introduce path graph, $P_N$, consisting of $N$ vertices and $N-1$ edges (Supplementary Fig. 4(a)). As mentioned in the main text, $\rho_N^{\rm sat}$ is calculated as follows:

$$\rho_N^{\rm sat} = \frac{1 + X_{N-3}}{N}. \tag{7}$$

The mathematical meaning of $X_N$ is the expected value of $N_{\rm O}$ for a path graph with $N$ vertices, $P_N$. Validity of this equation is confirmed as follows: All the vertices in a cycle graph $C_N$ are equivalent to each other, and any of them can be chosen as the first O-site. Since the selected O-site and the neighboring two vertices (N-sites) are not involved any longer in the succeeding packing process, we only have to consider the remaining part of the graph. The remaining subgraph is equivalent to a path graph $P_{N-3}$ because three successive vertices were removed, and the expected value of $N_{\rm O}$ for this part should be $X_{N-3}$ from the definition. Thus, the expected value of $N_{\rm O}$ for $C_N$ is $X_{N-3}+1$, and that should be equal to $N\rho_N^{\rm sat}$, from which we directly obtain Eq. (7). Note that the term of "+1" in the equation represents the firstly selected O-site in $C_N$.

Next, we prove that $X_N$ satisfies the following recurrence equation:

$$\begin{cases} X_0 = 0, \\ X_1 = 1, \\ X_2 = 1, \\ X_N = 1 + \dfrac{2}{N}\displaystyle\sum_{n=0}^{N-2} X_n. \end{cases} \tag{S3}$$

The cases for $N = 0, 1$ and 2 is obvious since the saturation states are uniquely determined (Supplementary Fig. 4(b)). For $N \geq 3$, one of the vertices is randomly chosen with an equal probability of $1/N$. We label the vertices as $n = 1, ..., N$ for convenience, where vertices 1 and $N$ are a terminal vertex (i.e., degree 1, see (Supplementary Fig. 4(a))). If a terminal vertex is chosen as the new O-site, a path graph $P_{N-2}$ is left as a remaining graph. In this case, the remaining subgraph is expected to have as many O-sites as $X_{N-2}$. Otherwise, if an internal (not a terminal) vertex $n$ ($2 \leq n \leq N-1$) is chosen, the remaining subgraph is two disjoint path graphs, i.e., $P_{n-2}$ and $P_{N-n-1}$. Note that $P_0$ is a null graph. Then, the remaining subgraphs have as many O-sites as $X_{n-2} + X_{N-n-1}$ in total. We can directly obtain the above recurrence equation by averaging the values over all the cases with a probability of

$1/N$, i.e., by taking a sum of all the cases and dividing by $N$:

$$\begin{aligned} X_N &= 1 + \frac{2}{N} X_{N-2} + \sum_{n=2}^{N-1} \frac{1}{N} (X_{n-2} + X_{N-n-1}) \\ &= 1 + \frac{2}{N} \sum_{n=0}^{N-2} X_n. \end{aligned} \tag{S4}$$

Note that the term of "+1" is corresponding to the operation to choose a vertex as the new O-site. By multiplying Eq. (S4) by $N$, we obtain

$$NX_N = N + 2 \sum_{n=0}^{N-2} X_n. \tag{S5}$$

Lastly, from the difference of $NX_N$ and $(N-1)X_{N-1}$, Eq. (8) is derived:

$$\begin{aligned} NX_N - (N-1)X_{N-1} &= \left( N + 2 \sum_{n=0}^{N-2} X_n \right) - \left( (N-1) + 2 \sum_{n=0}^{N-3} X_n \right) \\ &= 1 + 2X_{N-2} \end{aligned} \tag{S6}$$

$$\therefore X_N = \left( 1 - \frac{1}{N} \right) X_{N-1} + \frac{2}{N} X_{N-2} + \frac{1}{N}. \tag{8}$$

In summary, the saturation fraction of $N$-cycle graph, $\rho_N^{\mathrm{sat}}$, can be algebraically obtained in a recursive way by taking advantage of the fact that $N$-cycle is decomposed into smaller fragments (path graphs). Since the saturation state for path graphs can be exactly determined from a recurrence relation, $\rho_N^{\mathrm{sat}}$ for cycle graphs also can be obtained as well. It can be numerically confirmed that $X_N$ converges to $1 - e^{-2}$ with a damped oscillation-like behavior. Despite its apparent simplicity in the recurrence relation, there seems not to exist a simple exact explicit expression of $X_N$.

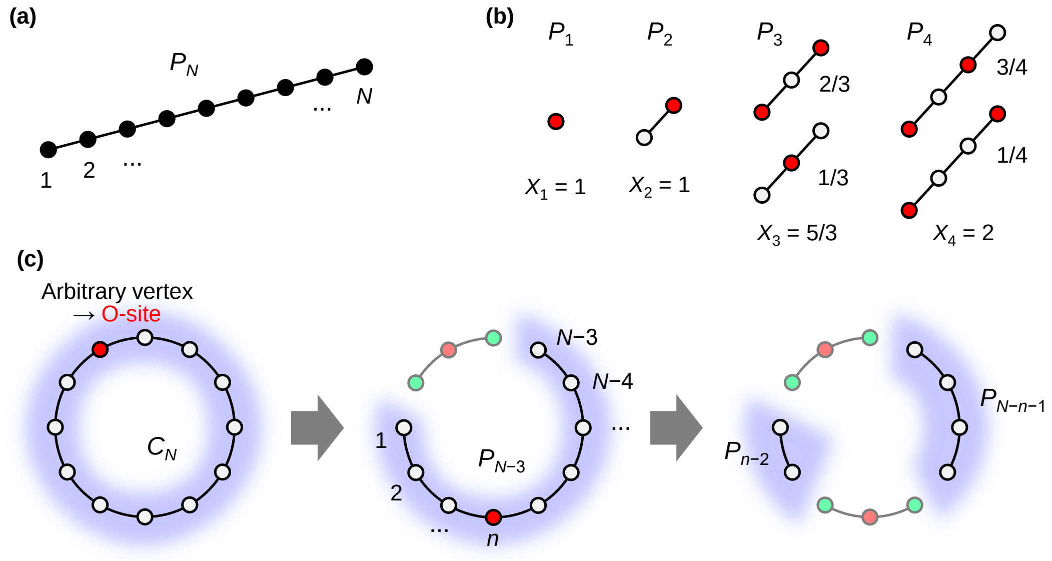


**Supplementary Figure 4** Schematic illustration of calculation procedure of $\rho_N^{\mathrm{sat}}$ for $N$-cycle graph based on path graphs. (a) Path graph $P_N$ with $N$ vertices. Vertices 1 and $N$ are a terminal vertex, whose degree is 1. Otherwise, vertex $n$ is adjacent with $n-1$ and $n+1$. (b) Packing pattern of small path graphs. A red vertex represents an O-site. Fractions accompanied with $P_3$ and $P_4$ indicate the probability that the corresponding pattern is realized. The expected value $X_n$ can be directly calculated from those figures for small path graphs. (c) Decomposition of cycle graph $C_N$ into path graphs. One vertex in the cycle graph $C_N$ is chosen as an O-site (left). Then, the remaining part is equivalent to a path graph $P_{N-3}$ (center). By repeating the packing process, the remaining part of the graph is decomposed into smaller path graphs (right).

# Supplementary Discussion 4: Random Packing Simulation Results for Simple Square Lattice with Neighboring Acceptance

We performed random packing simulations for the Sq lattice with a finite probability of accepting dopants adjacent to each other by introducing the weight parameter, as explained in Methods.

In the computational sense, the is no distinct difference between the A- and N-sites for $w > 0$ (i.e., finite neighboring acceptance), because they both can be selected as a potential dopant site. Rather they are only distinguished by difference in their weights. In addition, unlike the original model, there is no stopping criteria for packing simulation: Even after all the A-sites are filled by dopants, the N-sites still remain as potential dopant sites. After all, they are to be filled by dopants until all of them are occupied. Thus, it is required to define an *effective* number of the available sites which is consistent for arbitrary $w$, including the exactly non-neighboring case ($w = 0$) and complete random solution ($w = 1$). Here we defined the effective fraction of the available sites as $\rho_{\mathrm{A+N}} := \rho_{\mathrm{A}} + \sum_{i \in \text{N-site}} w_i$, where $w_i$ is the weight for neighboring acceptance for N-site $i$. It is easily confirmed that this definition satisfies the required condition.

Supplementary Figure 5 shows the snapshots obtained with increasing $\rho_{\mathrm{O}}$ for various $w$, where a certain fraction of dopant-dopant (O-O) neighboring observed. Supplementary Figure 6 shows the effective fraction of the available sites as a function of the O-site fraction. The extended model with $w > 0$ exhibits similar results to the original model ($w = 0$), and the original model result is regarded as the limit of $w \to 0$. Since there is no explicit stopping criterion in the case of $w > 0$, the saturation fraction $\rho_{\mathrm{sat}}$ cannot be uniquely determined. However, a clear change in the trend of $\rho_{\mathrm{A+N}}$ is found around $\rho_{\mathrm{O}} = \rho_{\mathrm{sat}}$ (especially in Supplementary Fig. 6b), which indicates that the saturation fraction is an essential characteristic to describe the behavior of repulsive dopant atoms even in the incomplete non-neighboring cases.

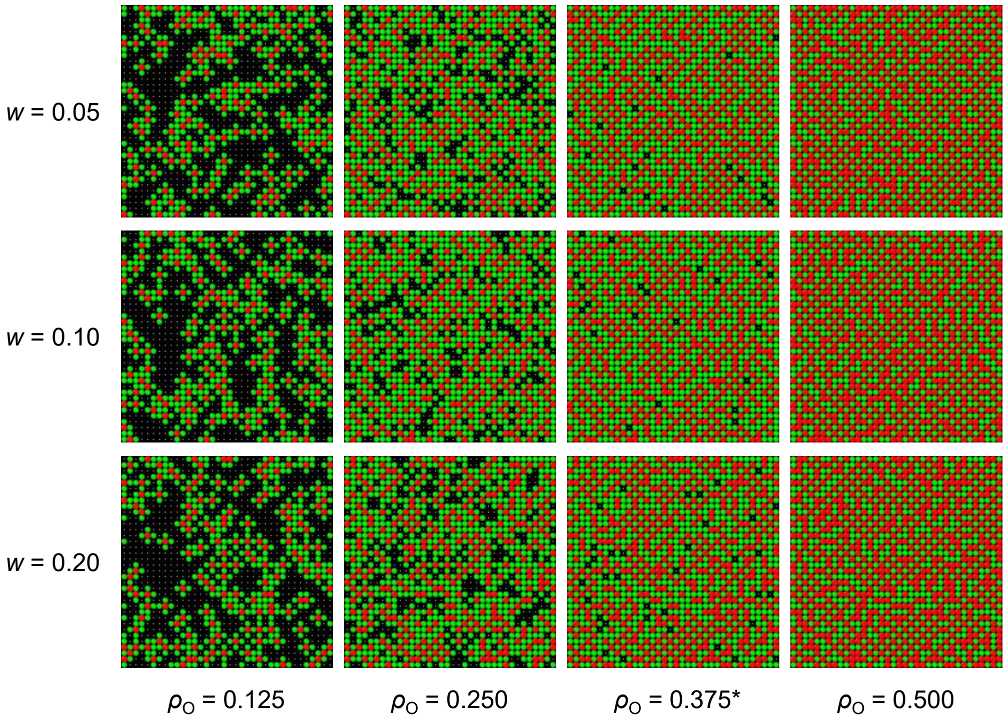


**Supplementary Figure 5** Snapshots of random packing for Sq lattice with finite probabilities of neighboring acceptance. A higher weight $w$ results in more acceptance of dopant-dopant neighboring.

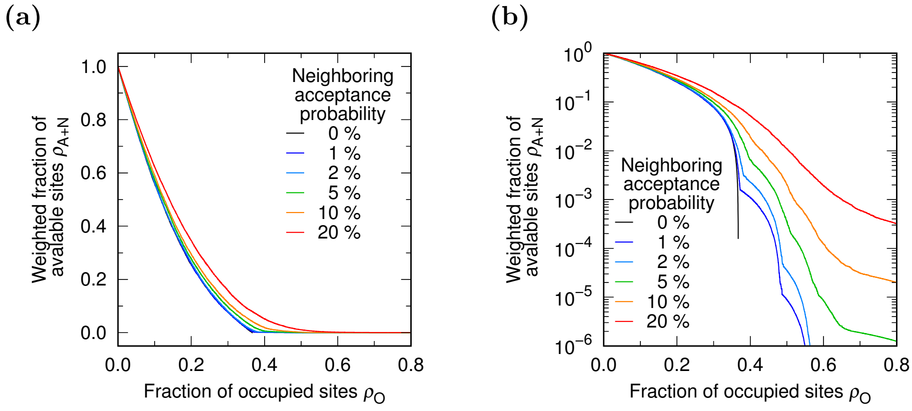


**Supplementary Figure 6** Effective available site fraction ($\rho_{\mathrm{A+N}}$) as a function of O-site fraction ($\rho_{\mathrm{O}}$) in (a) real number scale and (b) logarithmic scale for vertical axis.
