# Supplementary figures and images for: Graph-theoretic analyses of saturation fraction of repulsive dopants in solid solutions
Source: Sci Rep. 2026 Mar 12;16:7650. doi: 10.1038/s41598-025-30829-1 (PMC12982781; doi:10.1038/s41598-025-30829-1)

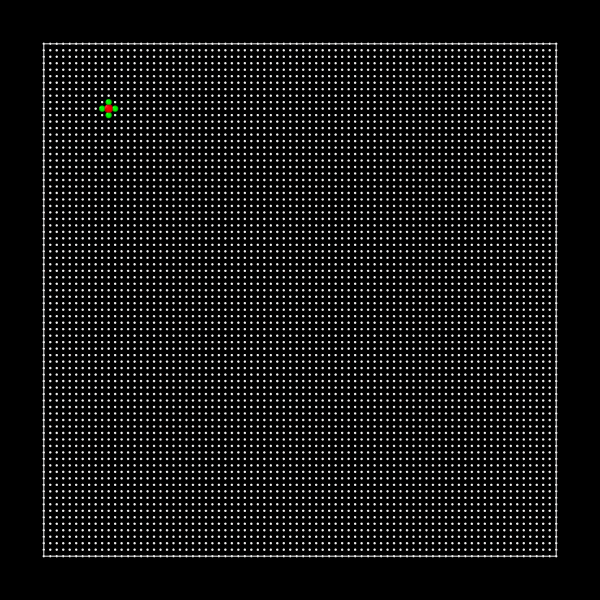

Supplement: Supplementary file 2 — Supplementary Information 2. [file 41598_2025_30829_MOESM2_ESM.gif]

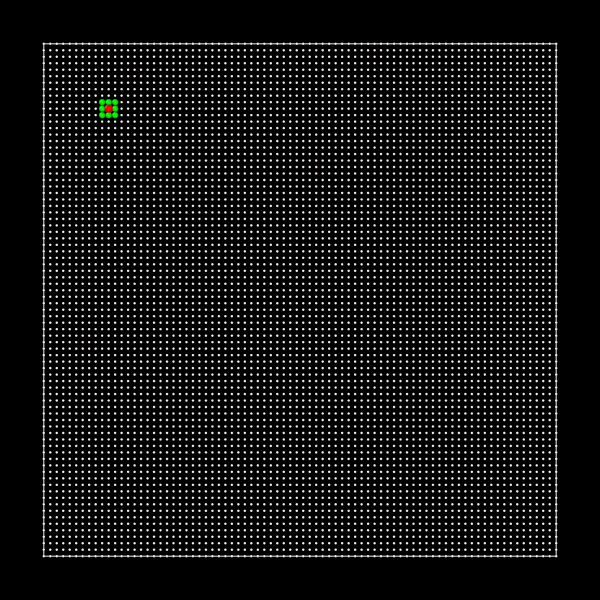

Supplement: Supplementary file 3 — Supplementary Information 3. [file 41598_2025_30829_MOESM3_ESM.gif]

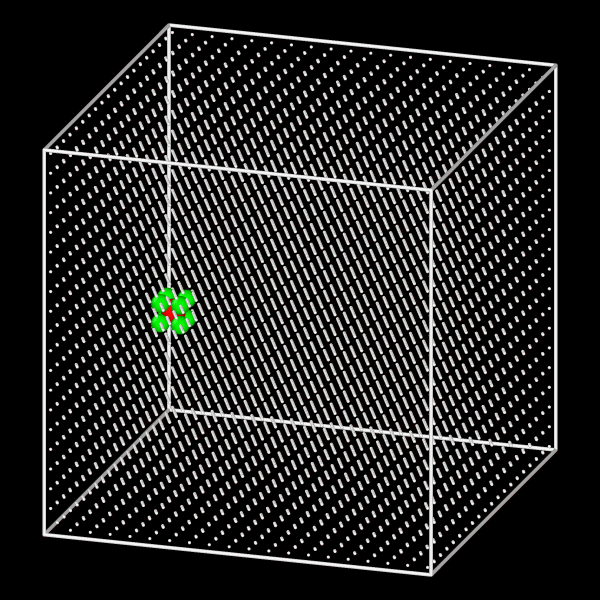

Supplement: Supplementary file 4 — Supplementary Information 4. [file 41598_2025_30829_MOESM4_ESM.gif]

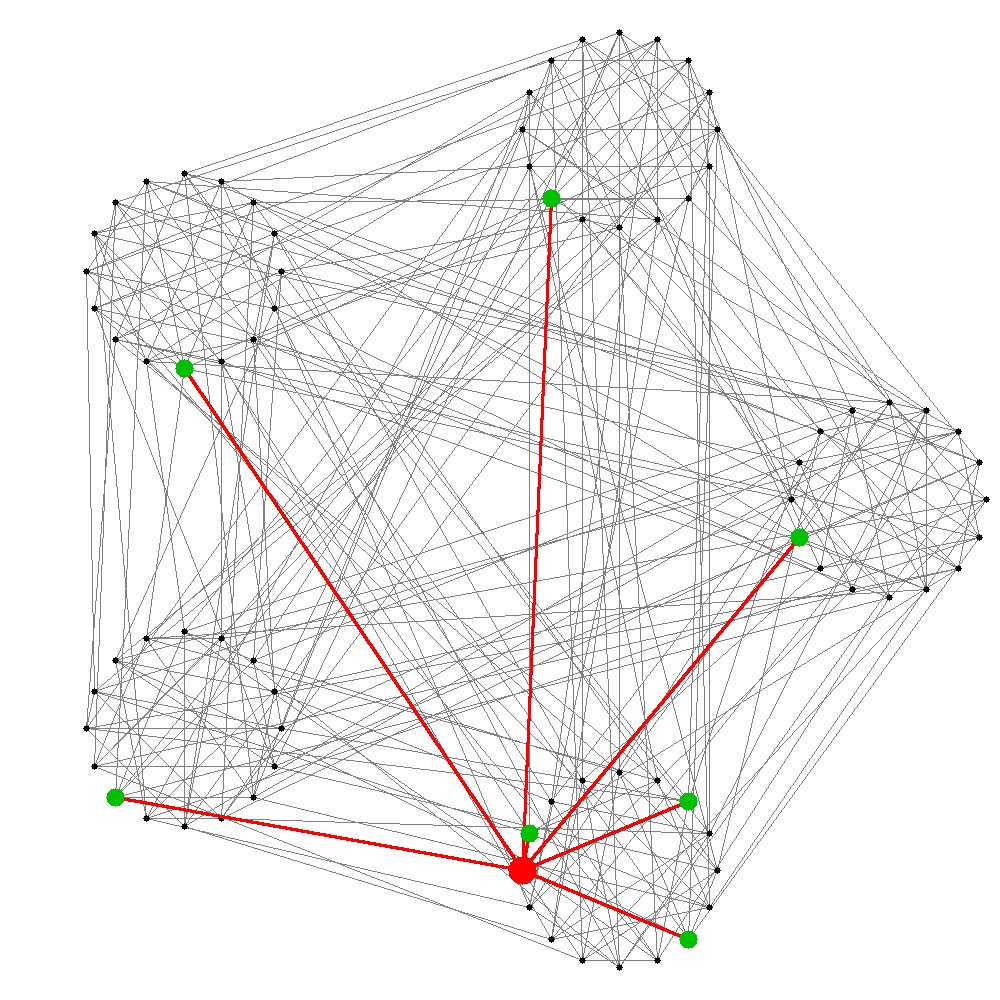

Supplement: Supplementary file 5 — Supplementary Information 5. [file 41598_2025_30829_MOESM5_ESM.gif]

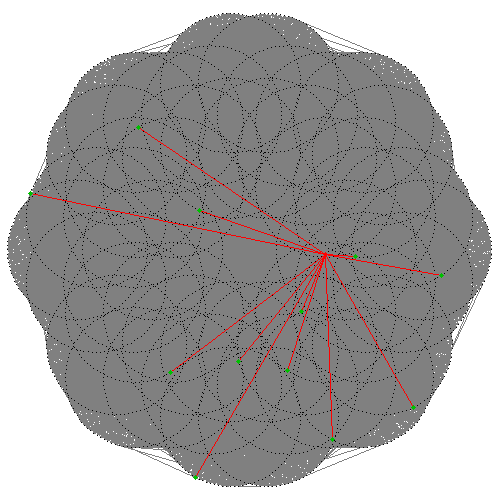

Supplement: Supplementary file 6 — Supplementary Information 6. [file 41598_2025_30829_MOESM6_ESM.gif]
